# Differences in clinical features observed between childhood-onset versus adult-onset systemic lupus erythematosus: A systematic review and meta-analysis

**DOI:** 10.1097/MD.0000000000008086

**Published:** 2017-09-15

**Authors:** Pravesh Kumar Bundhun, Alka Kumari, Feng Huang

**Affiliations:** a Institute of Cardiovascular Diseases; b Department of Obstetrics and Gynecology; c Institute of Cardiovascular Diseases and Guangxi Key Laboratory Base of Precision Medicine in Cardio-cerebrovascular Diseases Control and Prevention, the First Affiliated Hospital of Guangxi Medical University, Nanning, Guangxi, P. R. China.

**Keywords:** adult-onset, childhood-onset, clinical features, hematological manifestations, renal diseases, seizures, systemic lupus erythematosus

## Abstract

**Background:**

Systemic lupus erythematosus (SLE) affects people in childhood (childhood onset) or in adulthood (adult onset). Observational studies that have previously compared childhood-onset versus adult-onset SLE were often restricted to 1 ethnic group, or to a particular area, with a small sample size of patients. We aimed to systematically compare childhood-onset versus adult-onset SLE through a meta-analysis.

**Methods:**

Electronic databases were searched for relevant publications comparing childhood-onset with adult-onset SLE. Adverse clinical features were considered as the endpoints. The Newcastle Ottawa Scale (NOS) was used to assess the methodological quality of the studies and RevMan software (version 5.3) was used to carry out this analysis whereby risk ratios (RRs) and 95% confidence intervals (95% CIs) were used as the statistical parameters.

**Results:**

A total number of 10,261 participants (1560 participants with childhood-onset SLE and 8701 participants with adult-onset SLE) were enrolled. Results of this analysis showed that compared with childhood-onset SLE, pulmonary involvement was significantly higher with adult-onset SLE (RR: 1.51, 95% CI: 1.18–1.93; *P* = .001), whereas renal involvement was significantly higher with childhood-onset SLE (RR: 0.65, 95% CI: 0.55–0.77; *P* = .00001). Raynaud phenomenon and photosensitivity were significantly higher in adult-onset SLE (RR: 1.29, 95% CI: 1.04–1.60; *P* = .02) and (RR: 1.08, 95% CI: 1.01–1.17; *P* = .03), respectively. Malar rash significantly favored adult-onset SLE (RR: 0.84, 95% CI: 0.75–0.94; *P* = .002). Childhood-onset SLE was associated with significantly higher hemolytic anemia, thrombocytopenia, leukocytopenia, and lymphopenia. Seizure and ocular manifestations were significantly higher with childhood-onset SLE (RR: 0.57, 95% CI: 0.47–0.70; *P* = .00001) and (RR: 0.34, 95% CI: 0.21–0.55; *P* = .00001), respectively, whereas pleuritis was significantly higher with adult-onset SLE (RR: 1.45, 95% CI: 1.17–1.79; *P* = .0008). Vasculitis and fever were significantly higher with childhood-onset SLE (RR: 0.51, 95% CI: 0.36–0.74; *P* = .0004) and (RR: 0.78, 95% CI: 0.68–0.89; *P* = .0002) respectively.

**Conclusion:**

Significant differences were observed between childhood-onset versus adult-onset SLE, showing the former to be more aggressive.

## 1 Introduction

Autoimmune diseases have not well been studied through randomized controlled trials. However, even if small prospective, retrospective, and case–control studies were commonly used to study those diseases, they have gradually been able to show the impact of autoimmune diseases on the population.

Systemic lupus erythematosus (SLE) is one among the common autoimmune disorders affecting a large number of female patients.^[1]^ Even if it is often misdiagnosed or remains undiagnosed by physicians, several important classifications have been proposed according to recent guidelines.^[2]^ The diagnosis of SLE is based on 17 important criteria, whereby a diagnosis of SLE could be made based on 4 of the criteria, including at least 1 of the 11 clinical criteria and 1 of the 6 immunological criteria or by a biopsy-proven nephritis compatible with SLE in the presence of antinuclear antibodies (ANAs) or anti-double stranded DNA antibodies (ds-DNA).^[3]^

SLE affects people in childhood (childhood-onset) or in adulthood (adult-onset). However, observational studies that have previously compared childhood-onset versus adult-onset SLE were often restricted to 1 ethnic group,^[4]^ or to a particular area,^[5]^ with a small sample size of patients.^[6]^ Childhood-onset versus adult-onset SLE were not compared on an International or most probably on a worldwide basis (including patients from different parts of the world) to know whether the differences could be applied throughout any population. Therefore, we aimed to systematically compare childhood-onset versus adult-onset SLE using a large number of patients that were extracted from studies based on different regions, with different ethnic groups, in order to obtain a generalized outcome.

## 2 Methods

### 2.1 Data sources and searched strategies

Data sources included

(1) MEDLINE/PubMed database of medical research articles;
(2) EMBASE database;
(3) Cochrane library;
(4) www.ClinicalTrials.gov;
(5) Reference lists of suitable publications;
(6) Google scholar;
(7) Official websites of several journals of rheumatology.

### 2.2 Searched strategies

The following terms were used in the search process:

(1) “systemic lupus erythematosus,” “childhood,” and “adult”;
(2) “systemic lupus erythematosus” and “childhood”;
(3) “systemic lupus erythematosus” and “adult-onset”;
(4) “childhood-onset systemic lupus erythematosus”;
(5) “adult-onset systemic lupus erythematosus”;
(6) “childhood onset systemic lupus erythematosus” and “adult onset systemic lupus erythematosus.”

The abbreviation “SLE” was also used in this search process to replace its full-form.

Only English publications were searched.

### 2.3 Inclusion and exclusion criteria

Studies that satisfied the inclusion criteria were

(1) Studies that compared childhood-onset versus adult-onset SLE;
(2) Studies that reported clinical outcomes which were observed between childhood-onset versus adult-onset SLE;
(3) Studies that reported their data in the form of dichotomous data (number of events), which could be used in this analysis.

Studies were excluded based on the fact that

(1) They did not compare childhood-onset with adult-onset SLE;
(2) They did not report adverse clinical outcomes as their endpoints;
(3) They reported data in a form that could not be used in this meta-analysis;
(4) They were duplicate studies or replicated themselves through different searched databases.

### 2.4 Types of participants, outcomes, and definitions

This analysis involved participants with childhood-onset and adult-onset SLE, respectively. Onset of SLE before the age of 17 years was classified as childhood-onset, whereas SLE onset after the age of 17 years, but before the age of 50 years, was considered as adult-onset SLE in this analysis. Late-onset participants who acquired SLE after the age of 50 years were not included.

Endpoints that were assessed were first of all based on systemic involvement such as

(1) Pulmonary involvement;
(2) Gastrointestinal involvement;
(3) Dermatological involvement;
(4) Neurological involvement;
(5) Musculoskeletal involvement;
(6) Neuropsychiatric involvement;
(7) Renal involvement;
(8) Cardiovascular involvement;
(9) Hematological involvement;
(10) In addition, detailed clinical manifestations were also assessed.

### 2.5 Rheumatological and connective tissue manifestations

(1) Raynaud phenomenon
(2) Photosensitivity
(3) Alopecia
(4) Serositis
(5) Myositis
(6) Oral ulcers
(7) Arthritis
(8) Malar rash
(9) Discoid rash

### 2.6 Hematological manifestations

(1) Hemolytic anemia
(2) Thrombocytopenia
(3) Leukocytopenia
(4) Lymphopenia

### 2.7 Central nervous system manifestations

(1) Seizure
(2) Psychosis

### 2.8 Other clinical manifestations

(1) Pericarditis
(2) Ocular manifestations
(3) Pleuritis
(4) Vasculitis
(5) Fever.

The clinical features that were reported in each study have been summarized in Table 1.^[7–29]^

**Table 1:**
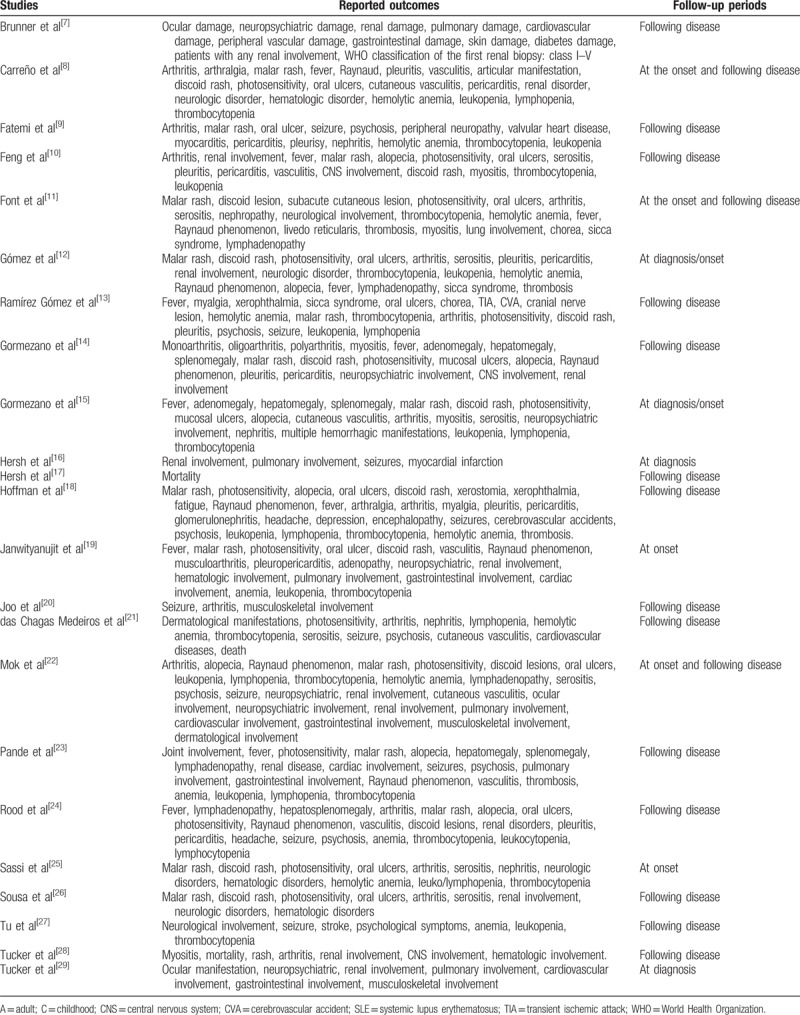
Types of participants, outcomes, and follow-up.

### 2.9 Data extraction and review

Data were extracted by 2 independent reviewers (PKB and AK). All the relevant information to be used in this analysis was collected. The clinical features that were reported, the age of disease onset, the types of participants, the total number of participants that were extracted from each study, the total number of events that were reported, were all recorded. As baseline features of the participants were seldom reported, we could not include these data in our analysis.

During this data extraction and data collection process, if ever any disagreement occurred, it was discussed between the 2 reviewers. However, if a final decision could not be made, the third reviewer (FH) was called to discuss and solve the issue.

As all the studies which were included in this analysis were observational studies, the Newcastle Ottawa Scale (NOS) was used to assess the methodological quality of the studies. NOS has been refined on the basis of expertise and experience whereby it was used in several projects.^[30]^

NOS assessment involved a minimum number of zero star to a maximum number of 9 stars depending on the quality of the study being assessed. The region where these studies were conducted and the number of stars allotted following the NOS assessment have been listed in Table 2.

**Table 2:**
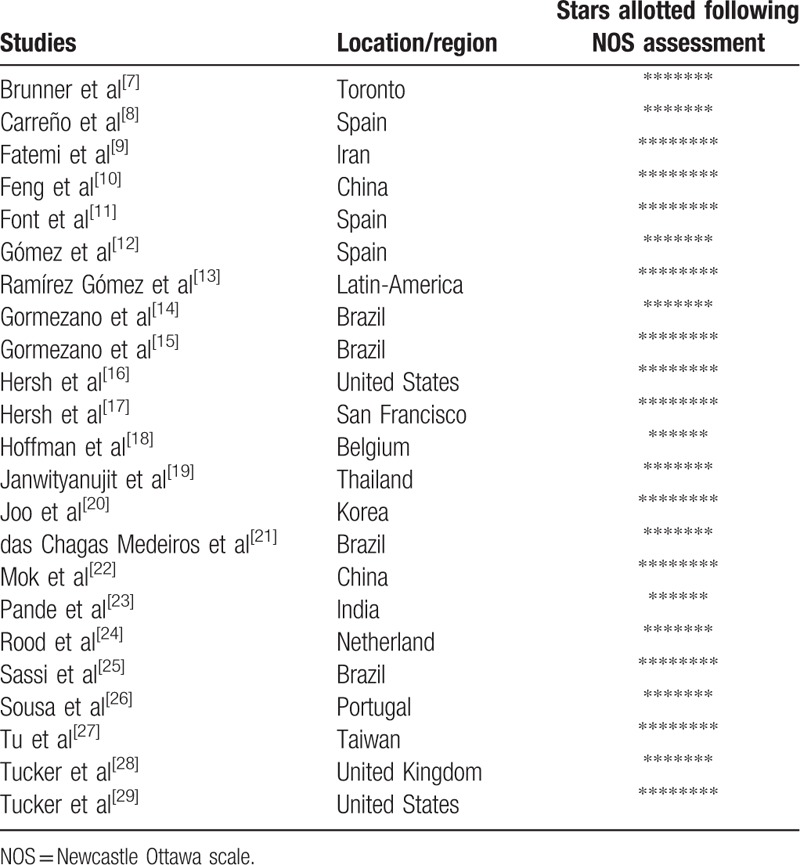
Study assessment using the Newcastle Ottawa Scale.

### 2.10 Statistical analysis

The latest version of the RevMan software (version 5.3) was used to carry out this analysis whereby risk ratios (RRs) and 95% confidence intervals (95% CIs) were used as the statistical parameters. However, a short coming that often affects meta-analyses is the presence of inconsistency across studies during subgroup analysis.^[31]^ Hence, the Q statistic test and the I^2^ statistic test were used to assess heterogeneity.

Statistically significant value was less or equal to 0.05.

Significance of *I*^2^: A low percentage of *I*^2^ denoted a low level of heterogeneity.

Fixed effects model: used if *I*^2^ was less than 50%.

Random effects model: used if *I*^2^ was greater than 50%.

Ethical approval was not necessary for this analysis.

Publication bias was visually assessed by observing funnel plots.

## 3 Results

### 3.1 Searched outcomes

The PRISMA study guideline was used.^[32]^ A total number of 1432 publications were obtained. A first elimination was directly carried out based upon assessment of the titles and abstracts whereby 1345 articles were rejected. Further eliminations were based on

(1) the study was a meta-analysis (1);
(2) the studies did not include any comparative group (14);
(3) the studies involved late-onset SLE participants (13);
(4) the studies were duplicates (36).

**Figure 1:**
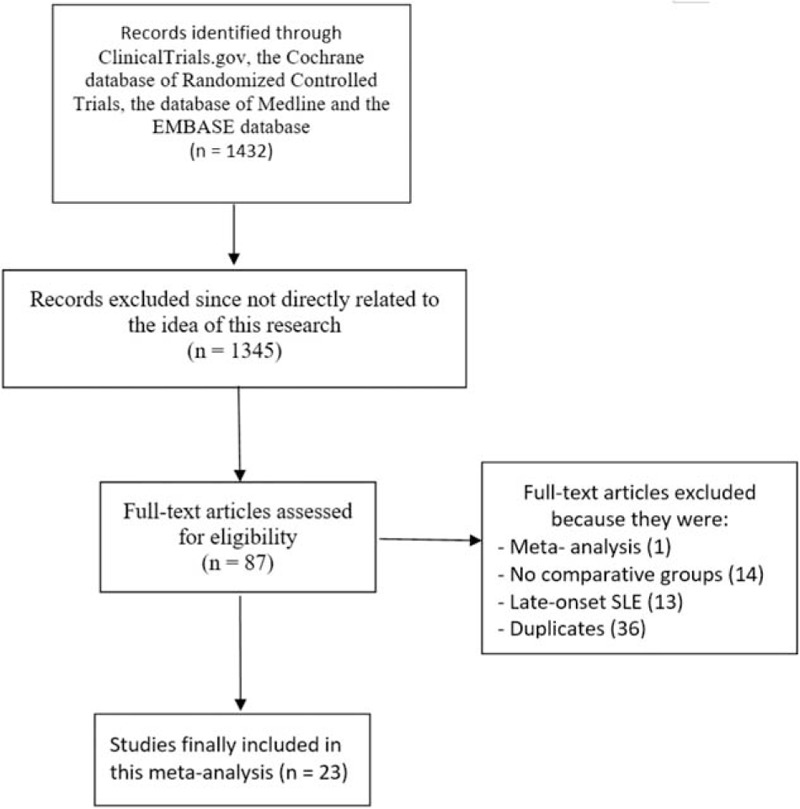
Flow diagram representing the study selection.

Finally, only 23 articles^[7–29]^ were selected for this analysis (Fig. 1).

### 3.2 Main features of the studies which were included

The types of study that were reported, the number of participants who were classified in the childhood-onset and the adult-onset SLE groups, respectively, and the time period of patients’ enrollment have all been listed in Table 3.

**Table 3:**
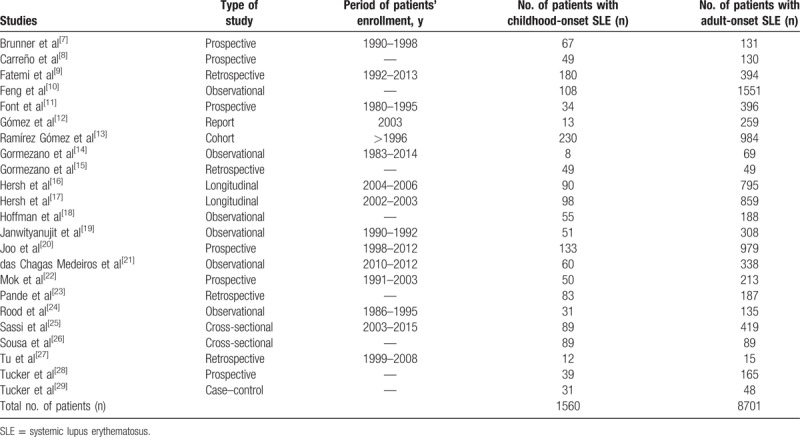
Main features of the studies which were included.

A total number of 10,261 participants (1560 participants with childhood-onset SLE and 8701 participants with adult-onset SLE) who were enrolled from the year 1980 to 2013 were included in this analysis.

### 3.3 System involvement

**Figure 2:**
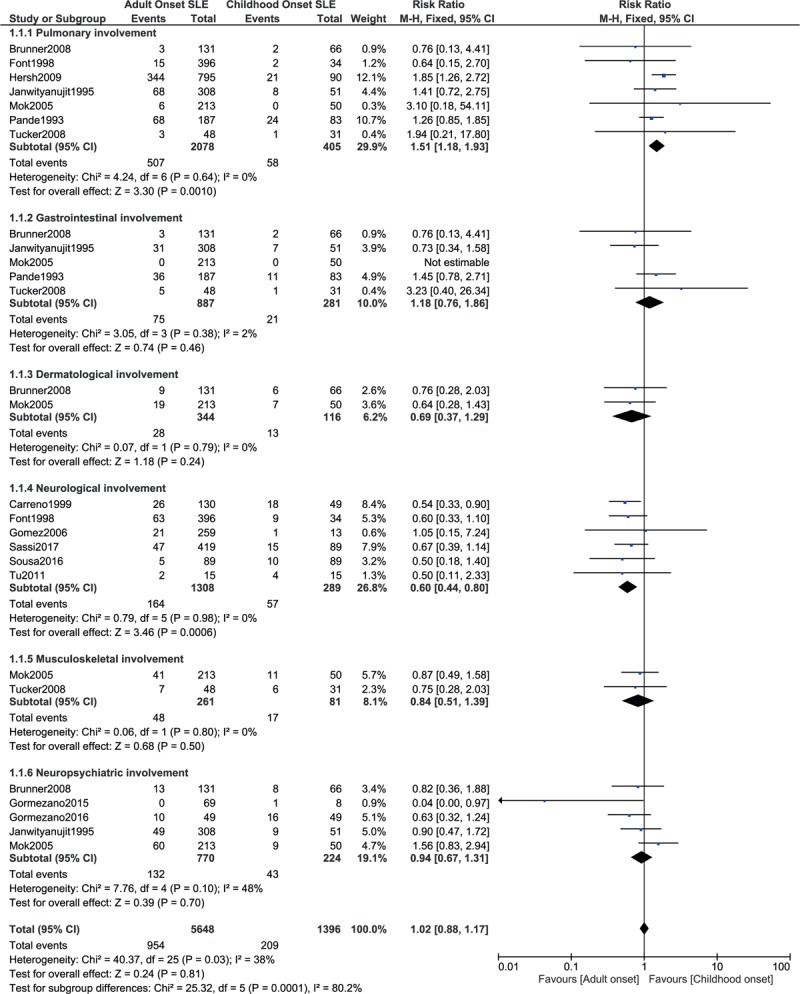
System involvement between childhood-onset versus adult-onset SLE (part 1).

Results of this current analysis showed that compared with childhood-onset SLE, pulmonary involvement was significantly higher with adult-onset SLE with RR: 1.51, 95% CI: 1.18 to 1.93; *P* = .001, *I*^2^ = 0% (Fig. 2). Gastrointestinal involvement, dermatological involvement, musculoskeletal involvement, and neuropsychiatric involvement as a whole were not significantly different between childhood-onset and adult-onset SLE with RR: 1.18, 95% CI: 0.76 to 1.86; *P* = .46, *I*^2^ = 2%, RR: 0.69, 95% CI: 0.37 to 1.29; *P* = .24, *I*^2^ = 0%, RR: 0.84, 95% CI: 0.51 to 1.39; *P* = .50, *I*^2^ = 0% and RR: 0.94, 95% CI: 0.67 to 1.31; *P* = .70, *I*^2^ = 48%, respectively, as shown in Fig. 2. However, neurological involvement was significantly higher in childhood-onset SLE with RR: 0.60, 95% CI: 0.44 to 0.80; *P* = .0006, *I*^2^ = 0% (Fig. 2). A fixed effects model was used to assess these outcomes.

**Figure 3:**
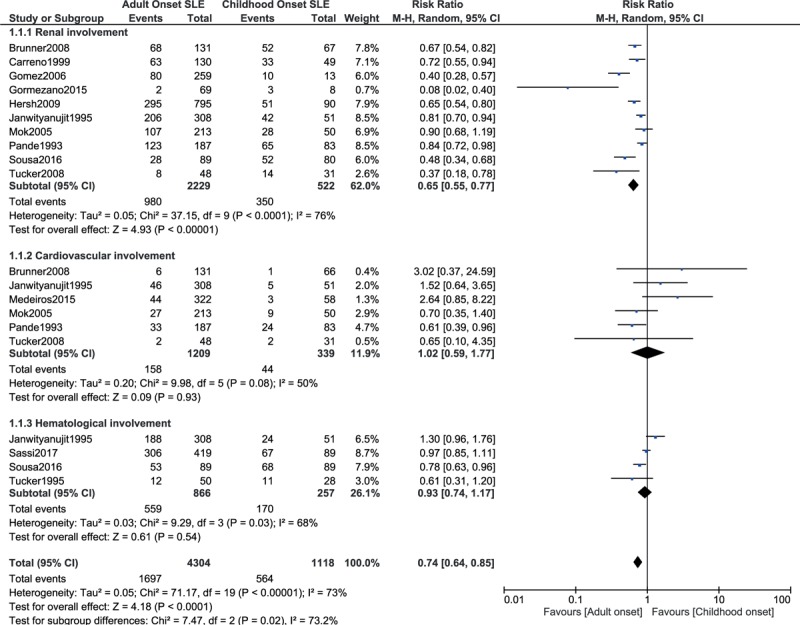
System involvement between childhood-onset versus adult-onset SLE (part 2).

A random effects model was used to assess several other outcomes. This analysis showed renal involvement to be significantly higher with childhood-onset SLE with RR: 0.65, 95% CI: 0.55 to 0.77; *P* = .00001, *I*^2^ = 76% as shown in Fig. 3. However, cardiovascular and hematological involvement as a whole were not significantly different with childhood-onset or adult-onset SLE with RR: 1.02, 95% CI: 0.59–1.77; *P* = .93, *I*^2^ = 50% and RR: 0.93, 95% CI: 0.74 to 1.17; *P* = .54, *I*^2^ = 68%, respectively (Fig. 3).

### 3.4 Rheumatological and connective tissue involvement

**Figure 4:**
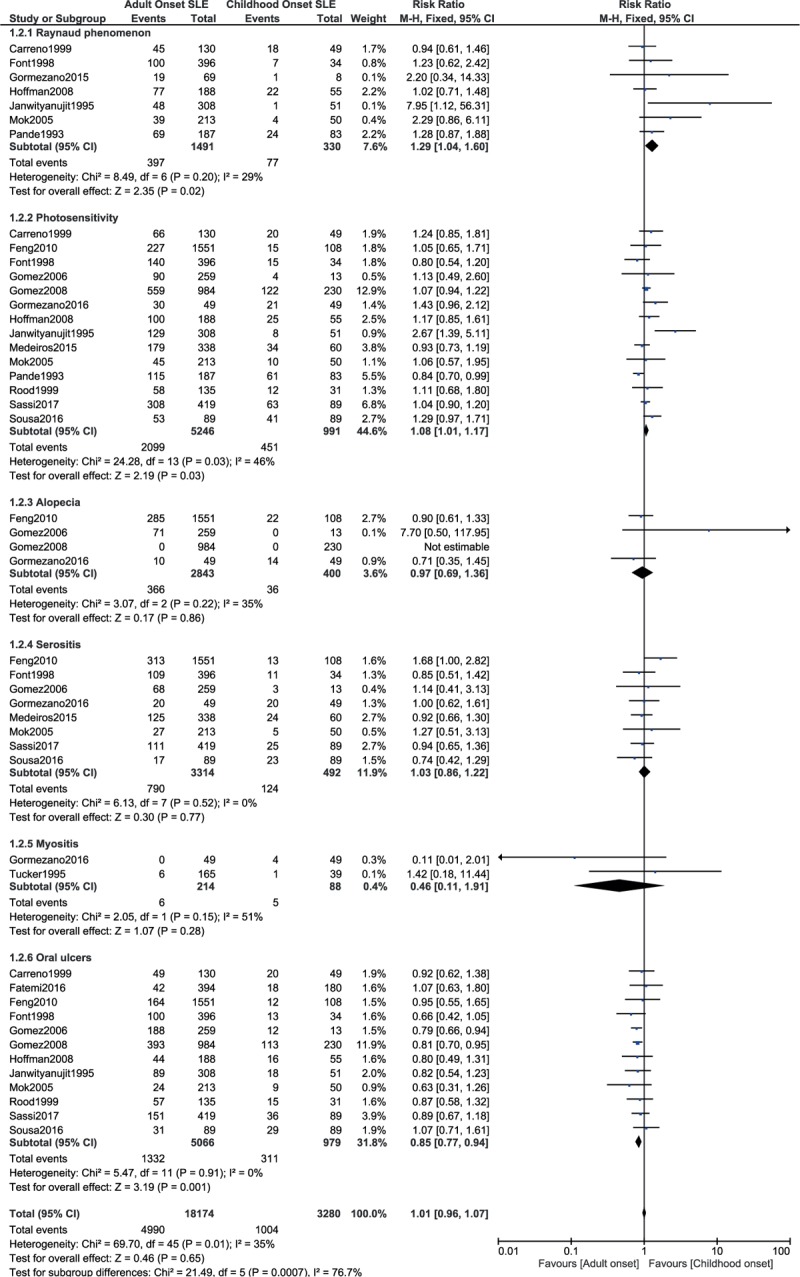
Rheumatological and connective tissue manifestations (part 1).

Raynaud phenomenon and photosensitivity were significantly higher in adult-onset SLE with RR: 1.29, 95% CI: 1.04 to 1.60; *P* = .02, *I*^2^ = 29% and RR: 1.08, 95% CI: 1.01 to 1.17; *P* = .03, *I*^2^ = 46%, respectively (Fig. 4). On the contrary, oral ulcers were significantly higher with childhood-onset SLE with RR: 0.85, 95% CI: 0.77 to 0.94; *P* = .001, *I*^2^ = 0% (Fig. 4). However, alopecia, serositis, and myositis were not significantly different with RR: 0.97, 95% CI: 0.69 to 1.36; *P* = .86, *I*^2^ = 35%, RR: 1.03, 95% CI: 0.86 to 1.22; *P* = .77, *I*^2^ = 0%, and RR: 0.46, 95% CI: 0.11 to 1.91; *P* = .28, *I*^2^ = 51%, respectively (Fig. 4).

**Figure 5:**
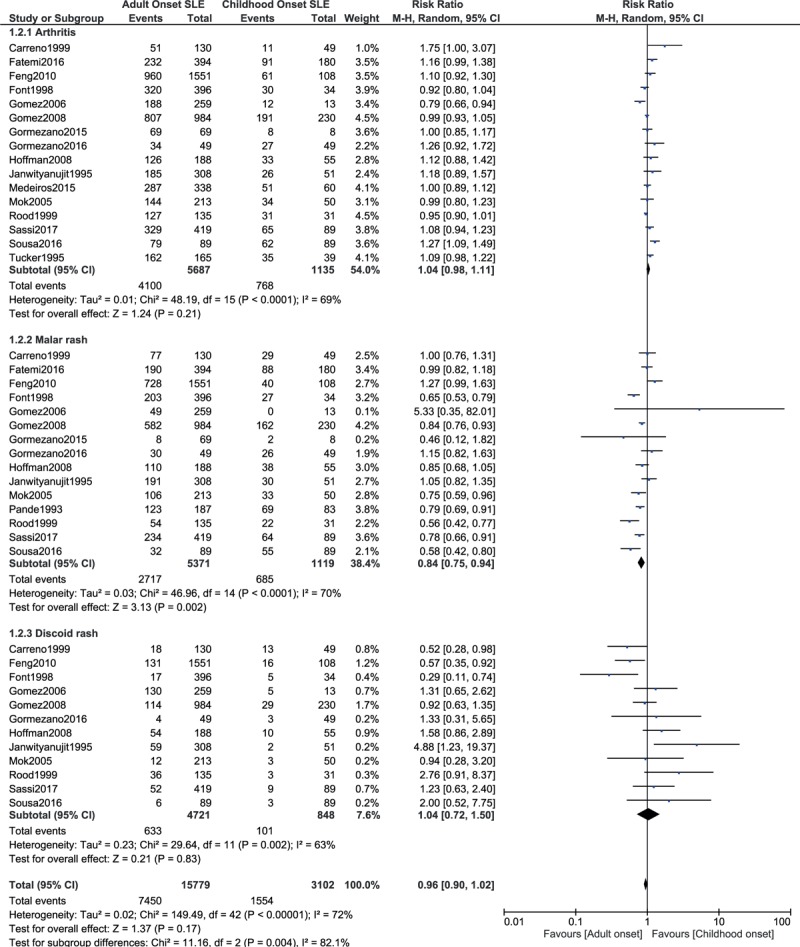
Rheumatological and connective tissue manifestations (part 2).

This current result also showed malar rash to significantly favored adult-onset SLE and affected patients with childhood-onset SLE to a higher extent with RR: 0.84, 95% CI: 0.75 to 0.94; *P* = .002, *I*^2^ = 70% (Fig. 5). However, arthritis and discoid rash were similarly manifested between childhood-onset and adult-onset SLE with RR: 1.04, 95% CI: 0.98 to 1.11; *P* = .21, *I*^2^ = 69% and RR: 1.04, 95% CI: 0.72 to 1.50; *P* = .83, *I*^2^ = 63%, respectively (Fig. 5).

### 3.5 Hematological manifestations

**Figure 6:**
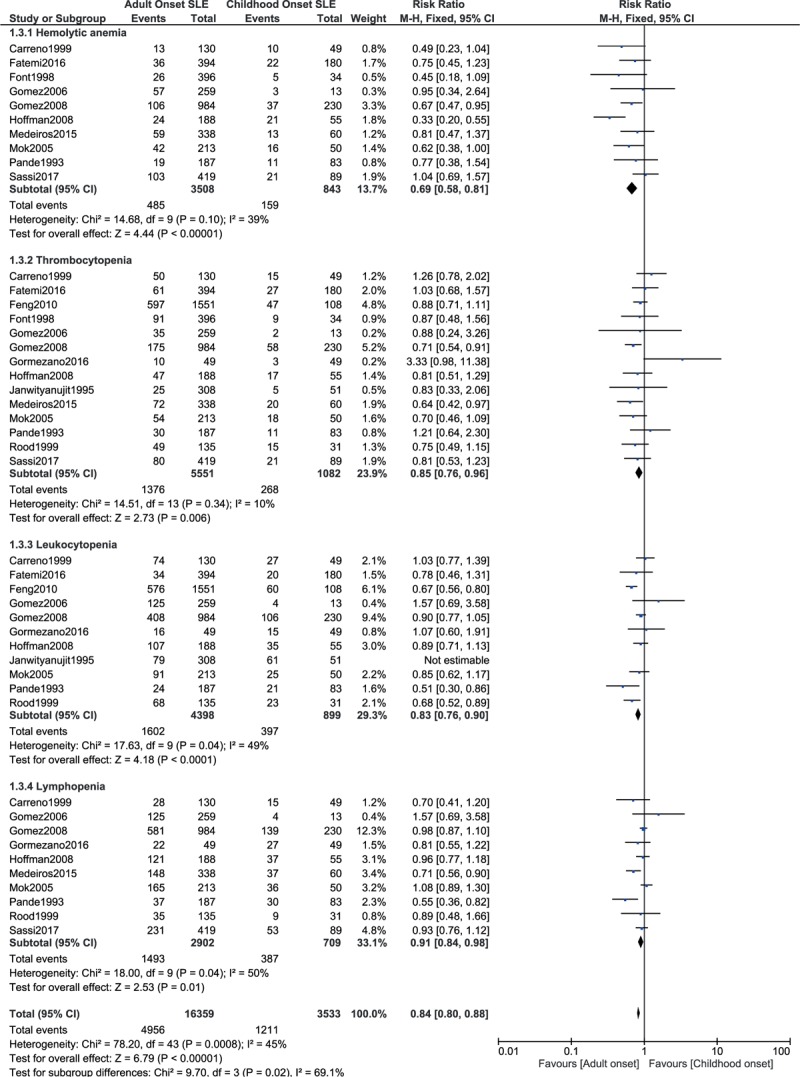
Hematological manifestations.

When hematological involvement was further subdivided, childhood-onset SLE was associated with significantly higher hemolytic anemia, thrombocytopenia, leukocytopenia, and lymphopenia with RR: 0.69, 95% CI: 0.58 to 0.81; *P* = .00001, *I*^2^ = 39%, RR: 0.85, 95% CI: 0.76 to 0.96; *P* = .006, *I*^2^ = 10%, RR: 0.83, 95% CI: 0.76 to 0.90; *P* = .0001, *I*^2^ = 49%, and RR: 0.91, 95% CI: 0.84 to 0.98; *P* = .01, *I*^2^ = 50%, respectively (Fig. 6).

### 3.6 Nervous system manifestations

**Figure 7:**
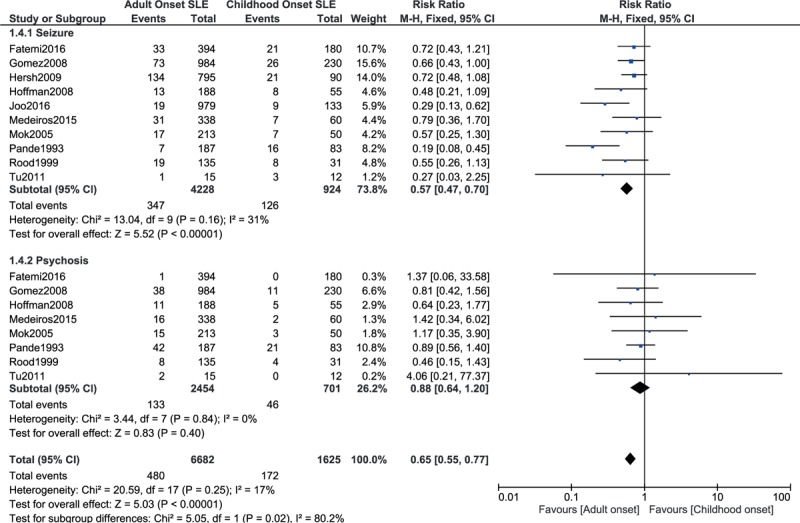
Nervous system manifestations.

Seizure was significantly higher with childhood-onset SLE with RR: 0.57, 95% CI: 0.47 to 0.70; *P* = .00001, *I*^2^ = 31%. However, no significant difference was observed with psychosis, with RR: 0.88, 95% CI: 0.64 to 1.20; *P* = .40, *I*^2^ = 0% (Fig. 7).

### 3.7 Other clinical manifestations

Ocular manifestation was significantly higher with childhood-onset SLE, with RR: 0.34, 95% CI: 0.21 to 0.55; *P* = .00001, *I*^2^ = 0%, whereas pleuritis was significantly higher with adult-onset SLE with RR: 1.45, 95% CI: 1.17 to 1.79; *P* = .0008, *I*^2^ = 0%. However, pericarditis was similarly manifested with RR: 0.84, 95% CI: 0.63 to 1.11; *P* = .23, *I*^2^ = 40%.

Vasculitis and fever were significantly higher with childhood-onset SLE, with RR: 0.51, 95% CI: 0.36 to 0.74; *P* = .0004, *I*^2^ = 53% and RR: 0.78, 95% CI: 0.68 to 0.89; *P* = .0002, *I*^2^ = 66%, respectively.

Significant and un-significant outcomes are listed in Table 4.

**Table 4:**
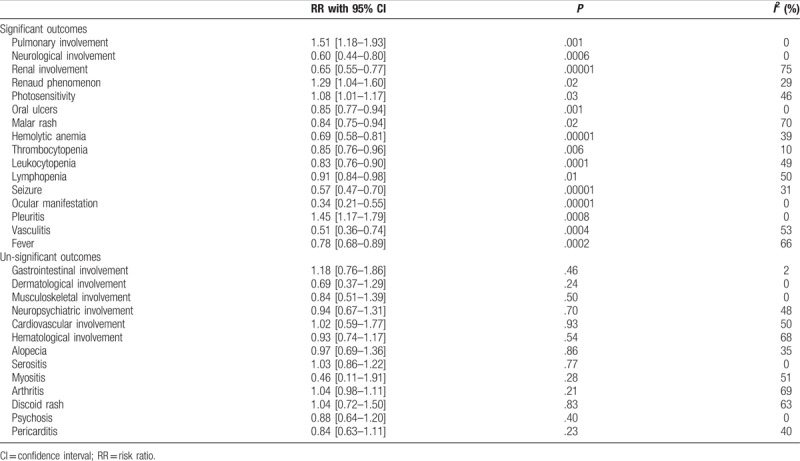
Results of this analysis.

### 3.8 Publication bias

**Figure 8:**
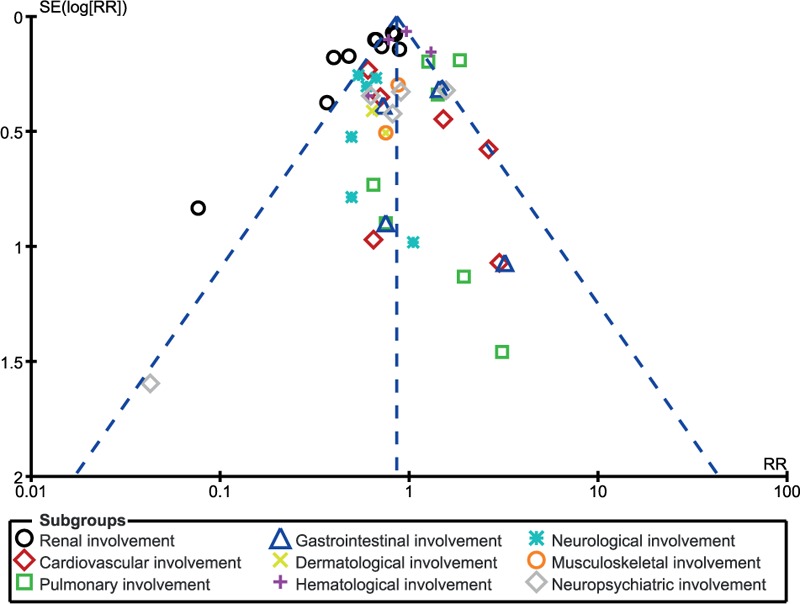
Funnel plot showing publication bias.

**Figure 9:**
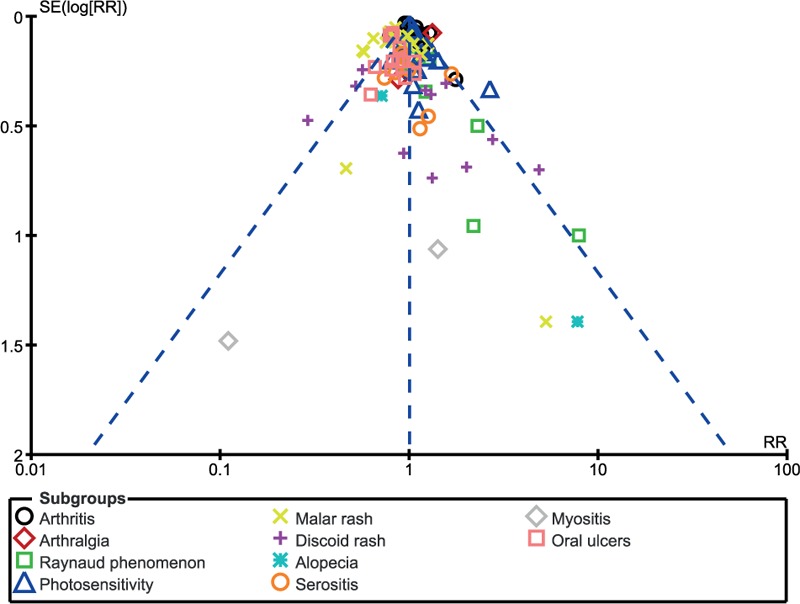
Funnel plot showing publication bias.

**Figure 10:**
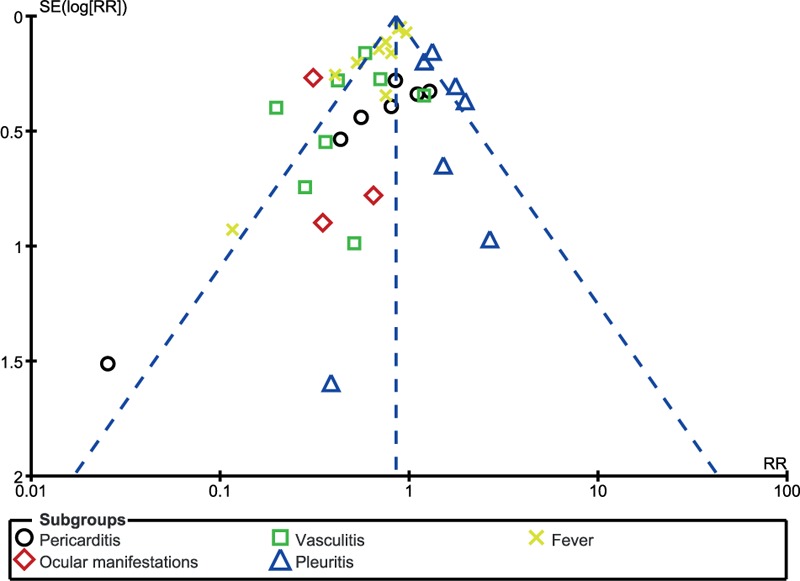
Funnel plot showing publication bias.

A visual assessment of the 3 funnel plots, which were obtained from RevMan, showed a low to moderate risk of publication bias across the studies that assessed the relevant clinical endpoints. The funnel plots have been represented in Figs. 8 to 10.

## 4 Discussion

In this analysis, we aimed to compare the clinical features that were associated with childhood-onset versus adult-onset SLE using a large number of participants, which was obtained from several corners around the world. The current results showed significantly more adverse features to be associated with childhood-onset SLE when compared with adult-onset SLE. Neurological and renal involvement were more significant with childhood-onset SLE. Even fever significantly favored adult-onset SLE compared with childhood-onset SLE. When hematological manifestation was further analyzed, hemolytic anemia, thrombocytopenia, leukopenia, and lymphopenia were significantly higher with childhood-onset SLE. However, pulmonary involvement, Raynaud phenomenon, and photosensitivity were significantly higher with adult-onset SLE.

A recent meta-analysis comparing the differences in autoantibody profiles and disease activity and damage score associated with childhood-onset versus adult-onset SLE showed increased anti-ds DNA and anticardiolipin antibodies to be significantly associated with childhood-onset SLE.^[33]^ The authors also suggested more disease activity in this category of SLE patients than adult-onset SLE. This current analysis has further supported their conclusion proving that more adverse clinical manifestations were present with childhood-onset SLE. Another meta-analysis comparing cutaneous manifestations between early-onset versus late onset SLE showed the latter to be associated with less severe outcomes.^[34]^ However, this current analysis did not involve patients with late-onset (elderly) SLE.

A review article based on the recent updates on the differences between childhood-onset and adult-onset SLE showed the latter to be 10 times more common than the former in United States. However, the authors mentioned that childhood-onset SLE was more severe.^[35]^ Another review article based on the similarities and differences between childhood-onset versus adult-onset SLE showed higher prevalence of renal involvement (nephritis) and central nervous system involvement in children than in adults, further supporting the results of this current analysis.^[36]^ The authors also suggested that additional steroid use and more aggressive treatment strategy should be considered in childhood-onset SLE. Moreover, data from the 2002 to 2010 cycles of the Lupus Outcomes Study showed childhood-onset SLE to significantly increase the risk of not working in adulthood, despite of full control of the disease.^[37]^

This current analysis showed childhood-onset SLE to be more aggressive; therefore, specific therapy with better management should be reserved to this particular subgroup. A few studies showed hematuria to significantly increase the mortality rate in participants with childhood-onset SLE that might have been due to complications associated with the renal organ.^[38]^ However, other studies have concluded that patients with childhood-onset and adult-onset SLE with renal involvement should both be carefully monitored to prevent unwanted outcomes.

This analysis satisfied all the criteria which are relevant for a good systematic review and meta-analysis. The methodological quality of the studies which were included were assessed. Robust results which match with the clinical literature were obtained. In addition, the current results have been generalized, and not limited to a specific ethnic group or region.

### 4.1 Novelty

This analysis is new because of several reasons:

(1) It is the first meta-analysis comparing clinical manifestations that were observed between childhood-onset versus adult-onset SLE; in contrast, a previously published meta-analysis only compared the laboratory features.
(2) This analysis includes a very large number of participants from different regions, thus, representing a generalized result that is not affected by a particular region or ethnic group.
(3) This idea is important in clinical medicine; the word SLE has often been heard, but, childhood-onset and adult-onset SLE, and their influence on clinical features might show something new to the readers.
(4) This analysis is very informative, showing a lot of data and results that are related to the differences between these 2 onset-periods of SLE, representing a new feature.

### 4.2 Limitations

This analysis also has several limitations:

(1) In those patients to whom clinical features were not reported following the course of the disease, clinical features at onset of the disease were considered relevant.
(2) All the data which were extracted were obtained from observational studies, which could be another limitation.
(3) Moderate to less severe heterogeneity was reported in several of the subgroups assessing the clinical features.

## 5 Conclusion

Significant differences were observed between childhood-onset and adult-onset SLE. Childhood-onset SLE was associated with significantly higher adverse clinical features whereby neurological involvement, renal involvement, oral ulcers, malar rash, vasculitis, fever, ocular, and hematological manifestations were significantly higher, whereas pulmonary involvement, Raynaud phenomenon, and photosensitivity were significantly higher with adult-onset SLE. However, no significant difference was observed in gastrointestinal involvement, cardiovascular involvement, discoid rash, psychosis, alopecia, serositis, and arthritis.
